# Whole pulse ingredient inclusion in macronutrient-balanced diets increased fecal concentrations of propionic acid but not total bile acids in healthy adult large-breed dogs after 20 weeks

**DOI:** 10.1093/jas/skaf075

**Published:** 2025-03-11

**Authors:** Pawanpreet Singh, Kelly S Swanson, Jennifer Saunders-Blades, Patricia M Oba, E James Squires, Anna K Shoveller

**Affiliations:** Department of Animal Biosciences, University of Guelph, Guelph, Ontario, CanadaN1G 2W1; Department of Animal Sciences, University of Illinois Urbana-Champaign, Urbana, IL, 61820USA; Champion Petfoods Holding Inc., Morinville, Alberta, CanadaT7X 2V9; Department of Animal Sciences, University of Illinois Urbana-Champaign, Urbana, IL 61820USA; Department of Animal Biosciences, University of Guelph, Guelph, Ontario, CanadaN1G 2W1; Department of Animal Biosciences, University of Guelph, Guelph, Ontario, CanadaN1G 2W1

**Keywords:** bile acids, canine, gut, pulses, fiber, starch

## Abstract

This study investigated the effects of up to 45% inclusion of whole pulse ingredients in grain-free (**GF**) diets on the excretion of bile acids (**BAs**) and other fecal metabolites in healthy large-breed dogs. Twenty-eight adult Siberian Huskies were fed 1 of 4 experimental diets formulated to meet the same macronutrient profiles for 20 wk: 1) grain-inclusive diet with 45% corn (Ctl), 2) GF diet with 15% pulses (Pulse15), 3) GF diet with 30% pulses (Pulse30), 4) GF diet with 45% pulses (Pulse45). All diets included chicken meal and pea starch. Fecal samples were collected on weeks 2 and 19. Bile acids were analyzed using ultra-performance liquid chromatography-MRM/MS technology, while fecal metabolites were analyzed using Agilent HP1000 high-performance liquid chromatography. Bile acids and fecal metabolite data were analyzed using the PROC GLIMMIX procedure in SAS studios (SAS version 9.4, SAS Inst., Inc., Cary, NC). All means were separated using the Tukey–Kramer adjustment (significant when *P* < 0.05). After 20 wk of feeding, concentrations of lithocholic acid were greater in Pulse15 and Pulse30 than Ctl (*P* = 0.001), but all were similar to Pulse45. Concentrations of deoxycholic (*P* = 0.054), lithocholic (*P* = 0.001), total secondary (*P* = 0.022), and total BA (*P* = 0.045) tended to be linearly associated with dietary pulse inclusion. Dogs consuming Pulse30 had greater fecal propionic acid concentrations than Ctl (*P* = 0.017), but both were similar to Pulse15 and Pulse45. Total branched-chain fatty acids (*P* = 0.001) and iso-butyric acid (*P* < 0.0001) were greater in Ctl than in all pulse groups. Inversely, arabinose concentrations were greater in all pulse groups compared to Ctl (*P* = 0.001). In summary, diets with up to 45% inclusion of whole pulse ingredients do not increase total BA excretion but may contribute to greater short-chain fatty acids production.

## Teaser

The inclusion of up to 45% whole pulse ingredients in extruded canine diets that exceed the American Association of Feed Control Officials’ minimum nutrient recommendations does not increase fecal bile acid concentrations but may contribute to greater fecal short-chain fatty acid and less branched-chain fatty acid concentrations. This suggests that pulse ingredients do not negatively impact bile acid recycling and may have positive effects on gut health in healthy large-breed dogs.

## Introduction

Pulses, such as peas, lentils, and chickpeas, are the dried seeds of legumes (FAO, 1994;Kaur et al., 2021), commonly used as a protein source in plant-based diets and a carbohydrate replacement for cereal grains in grain-free (**GF**) canine diets. GF diets are increasingly popular, constituting over 50% of the global dry pet food market (Choudhury, 2024). Pulses are low in fat (Tyler et al., 2017) and can provide up to 30% dietary protein (Boye et al., 2010). The amino acid (**AA**) profile of pulse ingredients complements that of cereal grains with higher concentrations of lysine but lower sulfur amino acids (**SAAs**), methionine, and cysteine (Mansilla et al., 2019; Templeman & Shoveller, 2022). Furthermore, pulses are rich in dietary fiber, particularly oligosaccharides. These complex carbohydrates resist enzymatic digestion in the small intestine, reaching the large intestine, where they support the growth of beneficial gut bacteria and the production of short-chain fatty acids (**SCFAs**; Holscher, 2017; Cai et al., 2019). As such, the composition of pulse ingredients supports myriad health benefits such as controlling glycemic response (Mitchell et al., 2009; McCrory et al., 2010), supporting weight management (Bosch et al., 2009; Reilly et al., 2020), sustaining immune function (Pinna & Biagi, 2014), and maintaining the intestinal barrier by contributing to anti-inflammatory metabolites that support gut health (Cockburn & Koropatkin, 2016).

However, the low methionine content in pulse ingredients has been speculated to play a role in taurine (**Tau**) deficiency, a potential risk factor for dilated cardiomyopathy in dogs (Fascetti et al., 2003; Backus et al., 2006; Ko et al., 2007; Kaplan et al., 2018). Taurine is a non-proteogenic SAA that is highly concentrated in cardiac muscle and plays a central role in systolic cardiac function (Kramer et al., 1981; Pion et al., 1987;Novotny et al., 1991; Gates et al., 2022), and bile acid (**BA**) metabolism (Bellentani et al., 1987). Pulse ingredients are not a dietary source of Tau, as Tau is only found in animal-based ingredients. Dogs can synthesize taurine endogenously, but the rate of biosynthesis can vary between breeds (Ko et al., 2007; Mansilla et al., 2020). These breed-specific differences and various dietary factors, such as the provision of dietary methionine, cysteine, and methyl donors, can impact whole blood, plasma, and urine Tau status in dogs (Tôrres et al., 2003; Kaplan et al., 2018; Banton et al., 2021). While the digestibility of indispensable AAs in pulse ingredients generally exceeds 80%, methionine is often less digestible (Johnson et al., 1998; Reilly et al., 2020). Conversely, in a recent study using the indicator AA oxidation technique, the bioavailability of methionine in ground dried peas was greater than in a specific batch of chicken meal when fed to large-breed dogs (Crosbie et al., 2024). These findings emphasize the substantial influence that processing can have on the variability of digestibility and bioavailability of AAs within intact ingredients. Nonetheless, there is substantial evidence indicating that the inclusion of pulse ingredients in GF canine diets that meet or exceed the minimum nutrient recommendations outlined by the Association of American Feed Control Officials (AAFCO, 2023), does not lead to a Tau deficiency, dilated cardiomyopathy, or other adverse health outcomes (Donadelli et al., 2020; Pezzali et al., 2020; Reilly et al., 2021; Leach et al., 2023; Singh et al., 2023; Banton et al., 2024; McCauley et al., 2024).

At the same time, the high oligosaccharide content in pulses may decrease enterohepatic recycling of BAs, potentially leading to increased fecal Tau losses (Hickman et al., 1992; Schneeman, 1999; Mansilla et al., 2019; Pezzali et al., 2021; Phungviwatnikul et al. 2022). BAs, synthesized from cholesterol, are typically conjugated with Tau or glycine in the liver. They then enter the small intestine, where they emulsify dietary fats, aiding in digestion and absorption (Russell & Setchell, 1992). Beyond their role in digestion, BAs act as signaling molecules involved in various metabolic pathways (Dawson & Karpen, 2015), and regulate cholesterol homeostasis, gut microbiota, gastrointestinal health, detoxification, and waste excretion (reviewed in Chiang, 2013). Primary BAs, cholic acid, and chenodeoxycholic acid constitute the majority of the endogenous BA pool. Secondary BAs, such as deoxycholic, lithocholic, and ursodeoxycholic acids, are produced by gut microbiota conversion from primary BAs and are present in lower amounts (Washizu et al., 1994; Hofmann, 1999). Approximately 95% of BAs are recycled in the ileum (reviewed in Chiang, 2013;van de Peppel et al., 2020); however as mentioned, greater soluble fermentable fiber in the diet can decrease BA recycling (Elhardallou, 1992), requiring the liver to synthesize more. Dogs preferentially conjugate BAs with Tau (Czuba and Vessey, 1981; Falany et al., 1994; Imamura et al., 2000), so a reduction in BA recycling could elevate Tau requirements, along with the demand for the precursor of Tau synthesis, cysteine.

Donadelli et al. (2020) reported that Labrador Retrievers had higher BA excretion, but increased plasma and whole-blood Tau after consuming a pulse-inclusive diet for 26 wk, compared with concentrations that followed 26 wk of consuming a commercial grain-based diet. However, in that study, there was no dietary control group and the macronutrient content between the treatment diet and baseline diet differed, making it difficult to isolate the effects of pulse ingredients on BA excretion. In contrast, a 28-d study with Beagles found no differences in BA excretion between dogs who consumed a GF pulse-inclusive compared to a pulse-free grain-inclusive diet (Pezzali et al., 2020). However, dogs on the pulse-inclusive diet had greater excretion of primary BAs, suggested by authors to be due to the higher dietary oligosaccharide content (Pezzali et al., 2020). It is important to note that the GF diet in that study also included potatoes and tapioca, which limited the ability to make conclusions specifically on the effects of pulse ingredients on BA excretion. Similarly, Beagles and mixed-breed hounds fed a low animal ingredient and GF diet with approximately 40% pulse inclusion had greater concentrations of primary BAs in their fecal samples compared to dogs consuming 1 of 2 grain-inclusive diets (Clark et al., 2023). This study included 4 dietary treatments, 2 grain-inclusive and 2 GF diets with either high or low animal protein inclusion (70% or 45% of total dietary protein came from a chicken-based ingredient, respectively). Dogs on the low animal GF diet with approximately 40% pulse inclusion, had lower concentrations of secondary BAs in fecal samples compared to the other 3 dietary groups. Notably, the high-animal GF diet contained significantly fewer whole pulse ingredients (approximately 3% green and yellow peas) compared to the low animal GF diet (approximately 40%), while having a higher proportion of pea starch (26.9%) and dehydrated potato flake (26.8%). Careful consideration of diet design, macronutrient content, and feed intake should be key criteria considered when comparing the literature on the inclusion of dietary pulse ingredients in GF formulations. Despite these differences, dogs on the GF 40% pulse-inclusive diet had comparable plasma and whole-blood Tau concentrations to those on grain-inclusive diets over the 180-d study period (Clark et al., 2023). Another study found no differences in BA excretion among dogs fed a rice-based, pea-based diet, or a rice diet supplemented with raffinose (an oligosaccharide), and no adverse effects on plasma or whole-blood Tau levels (Bokshowan et al., 2023). Similarly, previously published findings from this study (Singh et al., 2023; Banton et al., 2024) report no negative effects on whole-blood Tau, plasma Tau, or SAA status in dogs consuming GF diets with up to 45% pulse inclusion for 20 wk. On the other hand, dogs consuming a nutritionally deficient diet that did not meet the minimum AAFCO SAA recommendations and included wrinkled peas experienced compromised cardiac contractility and lower BA excretion than dogs consuming a nutritionally sufficient grain-based diet after 28 d (Quilliam et al. 2023). Evidently, there is limited data on how the inclusion of whole pulse ingredients alone, and their inherent characteristics in nutritionally adequate diets impacts BA metabolism and the production of various fecal metabolites in healthy adult large-breed dogs.

As such, this study investigated the effects of including up to 45% whole pulse ingredients in macronutrient-balanced diets that exceed the minimum AAFCO nutrient recommendations for adult dogs at maintenance on the excretion of BAs and concentrations of fecal metabolites such as those produced by microbes during fermentation (SCFAs, branch-chain fatty acids; **BCFAs**), and components of fiber that are released during fermentation (sugars such as lactose, glucose, arabinose, and xylose). We hypothesized that the fecal excretion of BAs and SCFAs would increase proportionally with the total dietary fiber and soluble: insoluble content in diets with higher whole pulse ingredient inclusion.

## Materials and Methods

### Animals and feeding

All experimental procedures were approved by the University of Guelph’s Animal Care Committee (Animal Use Protocol #4553). This study was conducted using a randomized control block design, from May to October 2021. Details about the diet and study design were previously described by Singh et al. (2023). In short, 28 outdoor-housed, healthy, adult Siberian Huskies (13 females, 4 intact; 15 males, 6 intact), ranging in age from 1 to 10 yr (mean age 5.29 ± 2.83 yr) and with an average body weight of 23.22 ± 3.73 kg were housed in outdoor free-run kennels (Rajenn Siberian Huskies, Ayr, Ontario, Canada). All dogs were deemed healthy before enrollment in the study based on complete blood count and biochemistry panels.

Four weeks before the start of the study period (weeks −4 to −1), all dogs were acclimated to being fed once a day at 1600 hours with a commercial extruded diet (Acana Healthy Grains Dog Food Red Meat, Champion Petfoods). Dogs were fed based on historical feeding records and had ad libitum access to fresh water. Throughout the study period, dogs were weighed weekly, and feed intake was adjusted to maintain baseline (week 0) BW. The mean feed intake in grams for each treatment group was: Ctl = 270.95 ± 23.72, Pulse15 = 327.98 ± 23.72, Pulse 30 = 328.3 ± 23.72, and Pulse45 = 291.51 ± 23.72.

### Diets and study design

On week 0, dogs were allocated to 1 of 4 extruded experimental diets (*n* = 7 per group) based on echocardiographic ejection fraction, age, body weight, and sex. The 4 experimental diets (Table 1) were formulated by Champion Petfoods Ltd. (Morinville, AB): 1) grain-inclusive diet with 33% corn (Ctl); 2) GF diet with 15% inclusion of whole pulse ingredients (Pulse15); 3) GF diet with 30% inclusion of whole pulse ingredients (Pulse30); 4) GF diet with 45% inclusion of whole pulse ingredients (Pulse45). All GF diets included a mixture of whole green and yellow peas, pinto beans, and a 50:50 blend of chickpeas and lentils. The range in pulse inclusion (0-45%) and macronutrient targets (30% crude protein, 13-15% crude fat) were meant to be representative of commercially available extruded diets in the pet food market. All diets included chicken meal and pea starch to balance protein and energy content. All diets were supplemented with the same micronutrient premix, used the same batch of ingredients, and were processed under similar conditions. No synthetic AAs were added to the experimental diets. Dogs were fed their respective diets for 20 wk. By provision of similar nutrient and ingredient delivery, we investigated whether any inherent properties of whole pulse ingredients impacted fecal BA or other fecal metabolite excretions.

**Table 1.** Ingredient composition and proximate analysis for experimental diets

Ingredient, %	Ctl	Pulse15	Pulse30	Pulse45
Whole grain corn	33	—	—	—
Corn gluten meal	12	—	—	—
Chicken meal	25	33	27.25	25
Pea starch	2.2	24.2	14.94	2.2
Whole green & yellow pea flour	—	5	10	15
Whole pinto bean flour	—	5	10	15
Whole chickpeas and lentil (50:50) flour	—	5	10	15
Fresh chicken	10	10	10	10
Chicken fat	7.5	7.5	7.5	7.5
Ground miscanthus grass	2	2	2	2
Natural chicken flavor (dry)	1.5	1.5	1.5	1.5
Natural chicken flavor (liquid)	2.5	2.5	2.5	2.5
Salt	2.5	2.5	2.5	2.5
Potassium chloride	0.75	0.75	0.75	0.75
Kelp	0.25	0.25	0.25	0.25
Proximate analysis (%, DMB)				
Moisture	9.58	10.6	11.2	10.2
Crude protein	35.30	36.60	35.50	36.10
Crude fat	17.10	15.10	15.30	15.90
Total dietary fiber	6.08	7.44	9.25	9.25
Nitrogen-free extract (NFE, calculated)^1^	37.41	35.23	35.71	34.33
Ash	7.84	9.64	9.80	9.84
Calculated metabolizable energy, kcal/kg^2^	3998	3797	3792	3816
Fiber, (% DMB)				
Soluble fiber	0.83	0.97	2.27	2.17
Insoluble fiber	5.25	6.47	6.97	7.08
Soluble:insoluble	0.16	0.15	0.33	0.31
Oligosaccharides, (mg/g DMB)				
Sucrose	5.42	12.16	13.99	13.46
Raffinose	0.51	3.49	4.40	2.77
Stachyose	0.90	9.30	8.96	11.47
Verbascose	0.36	5.18	3.95	3.30
Total starch (% DMB)	24.36	18.96	18.89	17.69

^1^NFE = 100-(moisture + protein + fat + fiber + ash).

^2^Metabolizable energy = ((8.5 kcal metabolizable energy (ME) x g crude fat) + (3.5 kcal ME × g crude protein) + (3.5 kcal ME × g nitrogen-free extract) × 10).

Composition of dietary treatments with increasing levels of whole pulse ingredient inclusion Ctl (0% inclusion), Pulse15 (15% inclusion), Pulse30 (30% inclusion), Pulse45 (45% inclusion) on an as-fed basis. DMB = dry matter basis.

All diets were formulated with equal inclusion of vitamin and mineral premixes; Vitamin B Premix Canine (0.2%), Vitamin ADE (0.2%), Choline Chloride (1.5%), Zinc (0.1%), Vitamin B5 (0.05%), Selenium (0.05%), Natural Antioxidant Liquid (0.04%), Natural Antioxidant Dry (0.02%), Copper (0.01%).

Technical additives per kg diet: Choline Chloride (0.4 ug), Copper proteinate (copper chelate of amino acid hydrate; 11 mg), Selenium yeast (0.3 mg), Vitamin A (15,000 IU), Vitamin D (2,000 IU), Vitamin E (200 IU), Vitamin B1 (100 mg), Vitamin B2 (40 mg), Vitamin B3 (200 mg), Vitamin B5 (57 mg), Vitamin B6 (30 mg), Vitamin B9 (3 mg), Zinc (Zinc chelate of amino acid hydrate; 160 mg).

### Fecal collection and analysis

Fecal samples were collected on weeks 2 and 19 and scored using a 5-point visual scaling system (1 = hard/dry, 5 = watery/diarrhea), where a score of 2.5 was considered ideal (Moxham, 2001). On both weeks, whole fecal samples were collected within 15 min of defecation and cleared of external contaminants such as grass, rocks or hair, then transferred into a Whirlpak bag (Thermo Fisher Scientific, Waltham, MA, USA) and kept on ice before being stored at −20 °C until analysis for fecal metabolites. On week 19, after 200 g of sample were collected per dog, subsequent collections for each dog were allocated into 4 separate 2mL cryovials, snap-frozen with liquid nitrogen on-site, and then stored at −80 °C until analysis for BAs.

Analysis of fecal metabolites, including SCFAs, BCFAs, glucose, arabinose, and xylose was conducted using the Agilent HP1000 high-performance liquid chromatography (**HPLC**) system (Agilent Technologies, Santa Clara, CA, USA). Samples were prepared and analyzed using the methodology described by Templeman et al. (2020).

Absolute quantitation of BAs was conducted using ultra-performance liquid chromatography-MRM/MS technology for high sensitivity by the Proteomics Centre (PC) at the University of Victoria (The Metabolomics Innovation Centre, University of Victoria, British Columbia, Canada). An internal standard was created by mixing targeted BAs at 10μM with a solution of 14 deuterated BAs. Fecal samples were dried by lyophilization and homogenized to a powder using a combination of a MM 400 mill mixer and 2 metal balls at 30Hz. Subsequently, 100 g of the powdered sample was transferred into 2 mL tubes and homogenized using a MM 400 mill mixer in a 70% acetonitrile solution at a rate of 10 μL per mg. The homogenization process was followed by sonication in an ice-water bath for a duration of 5 min and then centrifuged at 21,000 × *g* for 10 min at room temperature. After this, 20 μL of supernatant was diluted 10 times with the internal standard solution and 5 μL of this mixture was injected to run. Then, samples were further diluted 20-fold with the internal standard solution and then reinjected. All BAs were analyzed using an Agilent 1290 UHPLC system coupled to an Agilent 6495 QQQ mass spectrometer with the addition of an MS tool used in multiple-reaction monitoring with negative ion detection. Further detail on the operation parameters is described by Han et al. (2015).

### Statistical analysis

Fecal metabolites (SCFAs, BCFAs, lactose, glucose, arabinose, and xylose) were analyzed as repeated measures using the PROC GLIMMIX procedure in SAS (SAS version 9.4, SAS Inst., Inc., Cary, NC). BAs were analyzed for treatment effect, and the strength of linear, cubic, or quadratic relationships among treatments was analyzed using preplanned orthogonal contrast statements. For both models, dog was considered a random effect, and week and treatment (where applicable) were considered fixed effects. Significance was considered *P* < 0.05, and all means were separated using the Tukey–Kramer adjustment.

## Results

### Fecal scores, dry matter percentage, and fecal pH

The scores, dry matter percentage and pH of all fecal samples are reported in Table 2. Fecal scores were similar across treatment groups and time (*P* > 0.05). The average score of fecal samples was 3 (“moist beginning to lose form”). The dry matter percentage of all fecal samples was similar from treatments and over time (*P* > 0.05). The pH of samples from all treatment groups was lower in week 19 than in week 2 (*P* = 0.036); however, there were no treatment or treatment by time differences.

**Table 2.** Quality scores, dry matter % and pH of fecal samples from dogs consuming experimental diets with 0%, 15%, 30%, or 45% whole pulse ingredients (Ctl, Pulse15, Pulse30, Pulse45) after 2 and 19 wk, presented as least square means

	Week		Treatment				*P*-value		
	2	19	Ctl	Pulse15	Pulse30	Pulse45	TRT^1^	Week	TRT*Week
*n*	*28*	*28*	*7*	*7*	*7*	*7*			
Fecal scores	2.8	2.9	2.9	2.9	3.1	2.7	0.3813	0.152	0.6228
*n*	*28*	*24*	*14*	*13*	*14*	*11*			
Dry matter %	43.07	43.83	45.75	46.02	47.57	34.47	0.2891	0.8527	0.0927
Fecal pH	6.73^b^	6.54^a^	6.78	6.70	6.46	6.59	0.1014	0.0367	0.2019

^1^Treatment.

^a,b^Different letters within the same row indicate statistical significance (*P* < 0.05).

Fecal scores are based on a visual 5-point scale. The ideal score is considered 2.5 (1 = hard/dry, 5 = watery/diarrhea; Moxham, 2001). *n* = number of observations.

### Bile acids

There were no differences in total primary, secondary or overall BA concentrations among treatment groups (*P* > 0.05; Table 3). Dogs consuming Pulse15 and Pulse30 had greater lithocholic acid concentrations in fecal samples than Ctl (*P* = 0.001), but all were similar to Pulse45. Based on orthogonal contrasts, concentrations of deoxycholic (*P* = 0.054), lithocholic (*P* = 0.001), total secondary (*P* = 0.022), and total BAs (*P* = 0.045) displayed positive linear trends as pulse inclusion in the diets increased.

**Table 3.** Concentration of BAs in fecal samples from healthy adult dogs consuming 1 of 4 experimental diets with 0%, 15%, 30%, or 45% whole pulse ingredients (Ctl, Pulse15, Pulse30, Pulse45) after 20 wk of consumption, presented as least square means

Bile acid	Treatment											
(µmol/g)	Ctl	SEM	Pulse15	SEM	Pulse30	SEM	Pulse45	SEM	*P*-values			
*n*	*7*		*6*		*7*		*6*		TRT^1^	L	Q	C
Primary BAs^2^												
Cholic acid	0.149	0.140	0.147	0.156	0.121	0.108	0.182	0.192	0.992	0.920	0.998	0.791
Chenodeoxycholic acid	0.086	0.058	0.267	0.209	0.261	0.170	0.242	0.186	0.619	0.322	0.504	0.783
Total primary BAs	0.259	0.201	0.452	0.398	0.451	0.331	0.515	0.448	0.927	0.690	0.689	0.799
Secondary BAs												
Deoxycholic acid	3.304	1.066	8.213	2.962	6.403	2.140	4.062	1.465	0.261	0.054	0.951	0.774
Lithocholic acid	0.882^b^	0.231	3.694^a^	1.018	2.826^a^	0.709	1.812^ab^	0.498	0.008	0.001	0.408	0.935
Ursocholic acid	0.038	0.019	0.024	0.014	0.038	0.021	0.008	0.005	0.209	0.965	0.343	0.058
Total secondary BAs	4.701	1.382	12.966	4.199	10.074	3.013	6.431	2.082	0.131	0.022	0.922	0.814
Primary:Secondary	0.054	0.036	0.034	0.024	0.042	0.025	0.070	0.053	0.905	0.596	0.764	0.748
Total BAs^3^	5.384	1.689	14.372	5.106	11.831	3.901	8.451	3.002	0.209	0.045	0.711	0.986

^1^Treatment.

^2^Bile acids.

^3^Total BAs is summation of all BAs reported in this table and Table S1.

^a,b^Different letters within the same row indicate statistical significance (*P* < 0.05).

*n* = number of observations. Orthogonal contrasts; L = linear, Q = quadratic, C = cubic.

The total excretion of BAs, when reported as proportions, was similar among diets (*P* > 0.05; Table 4). There were also no differences in total primary BA excretions as a proportion to the total among treatment groups (*P* > 0.05). Proportions of fecal ursocholic acid were greater in the Ctl dogs compared to dogs consuming Pulse45 (*P* = 0.037), but both Ctl and Pulse 45 were similar to Pulse15 and Pulse30 (*P* > 0.05). Overall, there were no differences in the proportion of total secondary BAs excreted among treatment groups (*P* > 0.05). No relevant trends were observed in the proportions of BA excretions. Additional data on other BAs identified through the metabolomics analysis are available in the supplementary materials (Supplementary Tables 1 and 2), contributing to the limited information on the complete BA profile in healthy canines.

**Table 4.** Fecal bile acid proportions from healthy adult dogs consuming 1 of 4 experimental diets with 0%, 15%, 30%, or 45% whole pulse ingredients (Ctl, Pulse15, Pulse30, Pulse45) after 20 wk of consumption, presented as least square means

Bile Acid	Treatment											
*%, of total BA^1^	Ctl	SEM	Pulse15	SEM	Pulse30	SEM	Pulse45	SEM	*P*-values			
*n*	*7*		*6*		*7*		*6*		TRT^2^	L	Q	C
Primary BA, % of total												
Cholic acid	2.473	1.774	1.046	0.809	1.025	0.676	1.881	1.504	0.779	0.388	0.851	0.784
Chenodeoxycholic acid	1.602	0.737	1.930	0.940	2.228	0.931	2.531	1.296	0.911	0.849	0.563	0.802
Total primary acids	4.550	2.570	3.267	1.923	3.795	1.938	5.412	3.397	0.934	0.634	0.795	0.792
Secondary BA, % of total												
Deoxycholic acid	64.588	4.672	62.142	5.302	57.004	4.097	50.288	4.138	0.204	0.979	0.066	0.267
Lithocholic acid	15.065	3.213	24.599	5.751	24.571	4.830	21.219	5.072	0.421	0.175	0.482	0.962
Ursocholic acid	0.741^a^	0.348	0.167^ab^	0.076	0.330^ab^	0.137	0.098^b^	0.045	0.037	0.129	0.154	0.019
Total secondary acids	92.093	11.531	96.169	7.786	90.633	10.633	83.481	6.013	0.674	0.542	0.354	0.671
Primary:Secondary	0.052	0.035	0.034	0.023	0.041	0.024	0.073	0.054	0.887	0.595	0.734	0.711

^1^Bile acids.

^2^Treatment.

^a,b^Different letters within the same row indicate statistical significance (*P* < 0.05).

^*^Percentage derived from the total BAs presented in Table 3 and Table S1.

*n* = number of observations. Orthogonal contrasts; L = linear, Q = quadratic, C = cubic.

### Fecal metabolites

#### SCFAs and BCFAs

Fecal metabolite data is reported in Table 5. Treatment effects were observed for propionic acid (SCFA), iso-butyric acid (BCFA), and total BCFAs. Propionic acid concentrations were greater in the Pulse30 group compared to the Ctl group (*P* = 0.017), however, both groups were similar to Pulse15 and Pulse45 (*P* > 0.05). Both iso-butyric (*P* < 0.0001) and total BCFAs (*P* = 0.001) concentrations were greater in Ctl than in all pulse groups. There was a time effect observed for propionic acid (*P* = 0.007), total SCFAs (*P* = 0.043) and iso-butyric acid (*P* = 0.019), which were greater in week 19 compared to week 2. A treatment-by-week interaction was observed for total BCFAs (*P* = 0.004), where at week 2, concentrations of total BCFAs in all pulse-containing groups were lower than in the Ctl group. However, all groups were comparable to each other on week 19.

**Table 5:** Fecal short-chain fatty acid and branch-chain fatty acid concentrations of dogs fed experimental diets with 0%, 15%, 30%, or 45% whole pulse ingredients at weeks 2 and 19, presented as least square means

Fecal metabolites	Week			Treatment					*P*-value		
(umol/g)	2	19	SEM	Ctl	Pulse15	Pulse30	Pulse45	SEM	TRT^1^	Week	TRT * Week
Acetic acid	73.72	81.24	3.24	67.25	85.65	78.5	78.5	5.01	0.086	0.080	0.893
Propionic acid	34.55^b^	43.45^a^	2.27	29.8^b^	40.44^ab^	44.80^a^	40.93^ab^	3.33	0.017	0.007	0.934
Butyric acid	43.94	43.68	1.19	41.41	45.2	44.46	44.17	2.01	0.564	0.835	0.131
Formic acid	17.64	13.87	1.815	14.82	16.45	16.06	15.69	2.74	0.975	0.129	0.894
Iso-butyric acid	20.62^b^	22.27^a^	0.41	23.8^a^	20.27^b^	20.81^b^	20.72^b^	0.48	<0.0001	0.019	0.0630
Iso-valeric acid	2.74	2.44	0.22	2.89	2.77	2.91	1.81	0.22	0.280	0.424	0.488
Lactic acid	19.97	18.65	3.84	19.88	17.62	25.45	14.26	5.39	0.533	0.812	0.5149
Total SCFA^2^	152.12^b^	168.33^a^	5.91	138.52	171.00	167.78	163.60	9.07	0.057	0.043	0.870
Total BCFA^3^	22.44	23.35	0.43	25.51^a^	22.93^b^	21.91^b^	21.23^b^	0.66	0.001	0.126	0.004

^1^Treatment.

^2^Total SCFA = Acetic acid, Propionic acid, Butyric acid.

^3^Total BCFA = Iso-butyric acid, Iso-valeric acid.

^a,b^Different letters within the same row indicate statistical significance (*P* < 0.05).

Week 2 Ctl *n* = 6, Pulse15 *n* = 6, Pulse30 *n* = 3, Pulse45 *n* = 3.

Week 19 *n* = 2, Pulse15 *n* = 6, Pulse30 *n* = 3, Pulse45 *n* = 1.

#### Lactose, glucose, arabinose, xylose

Fecal arabinose concentrations were greater for all dogs who consumed a pulse-inclusive diet, compared to the grain-inclusive Ctl group (Table 6; *P* = 0.001). There were no other treatment differences among the concentrations of fecal sugar concentrations (*P* > 0.05). A time effect was observed for all monosaccharides (glucose, *P* = 0.032; xylose, *P* = 0.017; and arabinose, *P* = 0.0004) and lactose (*P* = 0.004). For all fecal samples, concentrations of all monosaccharides were lower in week 2 than in week 19 (*P* < 0.05), whereas concentrations of lactose were greater in week 2 than in week 19 (*P* = 0.004).

**Table 6:** Fecal monosaccharide (glucose, xylose, arabinose) and disaccharide (lactose) concentrations of dogs fed experimental diets with 0%, 15%, 30%, or 45% whole pulse ingredients at weeks 2 and 19 presented as least square means

Fecal sugar	Week			Treatment					*P*-value		
(umol/g)	2	19	SEM	Ctl	Pulse15	Pulse30	Pulse45	SEM	TRT^1^	Week	TRT * Week
Monosaccharides											
Glucose	5.03^a^	4.14^b^	0.46	3.79	4.17	4.9	5.37	0.75	0.462	0.032	0.369
Xylose	4.08^a^	2.84^b^	0.38	2.69	3.31	4.05	3.78	0.61	0.419	0.017	0.455
Arabinose	2.32^a^	1.56^b^	0.13	1.13^b^	1.97^a^	2.29^a^	2.36^a^	0.19	0.001	0.0004	0.053
Disaccharides											
Lactose	9.1^b^	10.85^a^	0.48	10.3	10.18	10.52	8.87	0.80	0.499	0.004	0.509

^1^Treatment.

^a,b^Different letters within the same row indicate statistical significance (*P* < 0.05).

Week 2 Ctl *n* = 6, Pulse15 *n* = 6, Pulse30 *n* = 3, Pulse45 *n* = 3.

Week 19 *n* = 2, Pulse15 *n* = 6, Pulse30 *n* = 3, Pulse45 *n* = 1.

## Discussion

Contrary to our hypotheses, there were no differences in total BA excretion across the diets, regardless of pulse inclusion and/or the soluble: insoluble fiber composition. The proportions of BAs observed in this study are comparable to previously reported ratios in healthy dog populations (Blake et al., 2019). Dogs consuming Pulse15 and Pulse30 had greater concentrations of lithocholic acid in their fecal samples than dogs on the Ctl diet. Similarly, dogs consuming Pulse30 had greater propionic acid concentrations than the Ctl group. Interestingly, the dogs in the Ctl group had similar concentrations of lithocholic and propionic acid to the group fed 45% pulses, despite differences in dietary pulse inclusion and/or total fiber content. These findings suggest that the amylose-to-amylopectin ratio in the diet may have had a stronger influence on the observed results than the total amount of pulse ingredients or fiber included.

In this study, dogs fed Pulse15 and Pulse30 diets had higher excretions of chenodeoxycholic acid, and consequently, greater excretion of lithocholic acid compared to Ctl. When primary BAs are not reabsorbed in the colon, they are metabolized by gut bacteria into secondary BAs through bile salt hydrolase activity, specifically 7α-dehydroxylases. As such, microbes convert chenodeoxycholic acid into lithocholic acid in the ileum (Fedorowski et al., 1979). Lithocholic acid is then primarily excreted in feces (Chiang, 2013). The increased lithocholic acid excretion observed in this study suggests enhanced conversion of chenodeoxycholic acid. This may be due to increased substrate availability for microbial conversion or higher levels of specific gut bacteria like *Clostridium* spp. (Ridlon et al., 2006; Yang et al., 2021) in the dogs consuming Pulse15 and Pulse30 diets. It is noteworthy that both the Ctl and Pulse45 diets had similar concentrations of pea starch (2.2%), while Pulse15 and Pulse30 contained much higher levels, at 24.2% and 14.94%, respectively. The pea starch used in the current study was separated from air-classified dehulled pea flour composed of approximately 70% starch, 7% to 10% crude protein, and 1% to 2% crude fiber. The processing method removed the fiber-rich hulls from the peas, which may explain why, despite higher concentrations of pea starch in Pulse15 and Pulse30, the total dietary fiber content is not significantly different between the experimental pulse-inclusive diets. While pea starch was added to help meet macronutrient targets and maintain consistent ingredient proportions across diets, pulse starches are known to have a high amylose-to-amylopectin ratio (Ren et al., 2021), which promotes the formation of resistant starch (Du et al., 2014). Rats fed high-amylose cornstarch diets had altered gut bacterial populations compared to rats provided with lower dietary amylose (Hu et al., 2022), which led to changes in BA metabolism (Hu et al., 2022) and increased fecal BA excretion (Kishida et al., 2002). Perhaps the pea starch inclusion in the current study facilitated higher dietary amylose and mediated BA excretion, separate from the effects of whole pulse ingredients, as observed for dogs consuming Pulse15 and Pulse30. Unfortunately, resistant starch was not analyzed in experimental diets and the microbiome was not measured in the dogs, which is a limitation to a deeper understanding of the mechanism. Indeed, the implications of dietary amylose and BA excretions in healthy adult dogs warrant further investigation. Nonetheless, the results of the present study were similar to another group of healthy dogs consuming a 45% green lentil diet, who had increased fecal lithocholic acid concentrations but no differences in total BA excretions compared to dogs on a pulse-free grain-inclusive diet (Reilly et al., 2021).

Furthermore, fecal ursocholic acid proportions were higher in the Ctl group compared to Pulse45, but both were similar to the other pulse-containing groups. Ursocholic acid is often degraded into deoxycholic acid and makes up a minor portion of the BA pool (Loria et al., 1986; Tint et al., 1992; Washizu et al., 1994), which may explain why, despite varying proportions between the Ctl and pulse-inclusive diets, ursocholic acid did not have a significant impact on total secondary BA excretion. Additionally, dogs consuming the Pulse15 diet had greater concentrations of sulfonated BAs than dogs consuming the Ctl diet, but both were similar to Pulse30 and Pulse45 (Table S1). Sulfonation is a phase II conjugation process that occurs following the conjugation of BAs with Tau or glycine (Sangaraju et al., 2021), where a sulfonate group is added to a functional component of the substrate, such as a hydroxyl or amine group (Alnouti, 2009). Sulfonation of BA reduces their toxicity and enhances their excretion via feces and urine (Alnouti, 2009). As such, the presence of pulse ingredients or pea starch may have beneficial implications for the detoxification of BAs. In humans, 40-75% of lithocholic acid is excreted in its sulfonated form (Alnouti, 2009), and although dogs have lower sulfation capacities than humans (Sangaraju et al., 2021), the higher lithocholic acid concentrations in the Pulse15 group likely contributed to the overall concentration of sulfonated BAs in the fecal samples.

As hypothesized, dogs consuming the Pulse30 diet exhibited greater excretion of propionic acid compared to dogs consuming the Ctl diet; however, the concentrations of propionic acid in both groups were similar to the Pulse15 and Pulse45 groups. A similar increase in SCFA production was observed in the same group of dogs when supplemented with similar dietary soluble: insoluble fiber content to that in the current study, over a 9-wk period (Thornton et al., 2021). On the other hand, dogs consuming the grain-based Ctl diet had greater concentrations of BCFAs. The production of BCFAs is typically negatively associated with protein digestibility, as the gut microbiota utilize dietary branched-chain AAs to synthesize BCFAs, such as iso-butyric acid (Taormina et al., 2020). Thus, BCFA production is often considered a result of increased proteolytic fermentation, which can produce metabolic byproducts that contribute to inflammation, increased gut permeability, and colitis, adversely impacting gut health (Diether & Willing, 2019). Overall, the digestibility of indispensable AAs in all diets, as measured by the cecectomized rooster assay, exceeded 75% (Banton et al., unpublished). In addition, at week 20, all groups had comparable concentrations of BCFAs. Thus, the difference in BCFA concentrations is not likely associated with differences in protein digestibility. Similar concentrations of total BCFAs were observed in a healthy population of mixed-breed hounds consuming diets with approximately 30% protein that were grain-inclusive, or GF and pulse-inclusive, where protein digestibility exceeded 80% in all diets as assessed through apparent total tract digestibility (Clark et al., 2023). Unlike in the current study, hounds on the GF pulse-inclusive diet had higher BCFA concentrations on day 180 compared to those on grain-inclusive diets, which authors speculate to be due to fecal microbiota—however, the mechanism is not delineated (Clark et al., 2023). However, on days 30, and 90, the BCFA concentrations in fecal samples were lower than in the grain-inclusive groups, aligning with the findings of the present study. Moreover, in agreement with the current study, dogs consuming diets with greater fiber and similar soluble:insoluble fiber content to the experimental pulse diets were reported to have lower fecal concentrations of BCFAs (Montserrat-Malagarriga et al., 2024). More data is needed to support that whole pulse ingredients contribute to greater total SCFA production, which would prove to have myriad health benefits such as supporting immune function, energy metabolism, cardiovascular health, etc (reviewed in, Xiong et al., 2022). In the current study, total dietary fiber content was not controlled but instead a direct result of the inclusion of whole pulse ingredients, with Pulse45 containing 9.25% total dietary fiber (dry matter basis). In contrast, diets in the Clark et al. (2023) study were formulated to maintain a consistent total dietary fiber level (approximately 15% on a dry matter basis). Despite this, authors report the highest SCFA concentrations in fecal samples from hounds consuming the GF diet with approximately 40% whole lentil inclusion compared to the GF diet with approximately 3% whole lentil inclusion and 2 grain-inclusive diets. Similar to the current study, both the GF diets contained high levels of pea starch, with the GF 40% lentil-inclusive diet containing 19.5% and GF 3% lentil-inclusive diet containing 26.9% pea starch (Clark et al., 2023). Together, findings from both studies suggest that whole pulse ingredients—not just pea starch—play a key role in SCFA production.

All dogs in this study had higher fecal concentrations of SCFAs with a corresponding decrease in monosaccharide concentrations in week 19 compared to week 2. The gut microbiota primarily utilizes carbohydrates from dietary fiber breakdown like monosaccharides including glucose, arabinose, and xylose for the production of SCFAs (Holscher, 2017; Williams et al., 2017). In the current study, fecal samples of dogs consuming pulse-inclusive diets had greater concentrations of arabinose than dogs consuming the Ctl diet. This may be attributed to the higher insoluble fiber content in pulse ingredients, due to the presence of hemicellulose, which is composed of xylose and arabinose (Lavarack et al., 2002;Siva et al., 2019). However, it is difficult to interpret data regarding fecal sugars without knowing the quantity of each fiber fraction in the diets and how much of that intact fiber remained in the feces. Regardless, there was greater saccharolytic fermentation for all diets as elucidated by higher SCFA concentrations in week 19 than in week 2. This change in all diets may be attributed to the wash-in diet (Healthy Grains Dog Food Red Meat, Champion Petfoods) used in this study having lower fiber, and greater fat than the experimental diets. Furthermore, all fecal samples had a lower pH at week 19 than week 2. Higher concentrations of glucose and xylose—byproducts of fiber breakdown—along with the presence of SCFA, can contribute to a lower gut pH, which subsequently lowers the pH of fecal samples (Jensen et al., 2024). The pH of fecal samples for all dogs in the current study is representative of a healthy population (Brambillasca et al., 2010). Additionally, it is important to highlight that the fecal scores for all experimental groups were not different from each other throughout the study period and were considered ideal.

The results of this study are limited to this specific combination of pulse ingredients in the presence of these macronutrient profiles and processing methods. Additionally, the variability of pea starch content across diets may confound the results, limiting conclusions about the impact of whole pulse inclusion on fecal excretion of BAs and other metabolites. Additionally, the impact of pulse inclusion in GF diets on the gut microbial populations of healthy dogs remains a knowledge gap that must be addressed to better understand the role of these ingredients on BA metabolism. Unfortunately, gut and fecal bacterial populations were not assessed in this study. Moreover, the small sample size in each dietary treatment is a limitation, particularly due to the high variation in gut microbiome composition between individual dogs (You & Kim, 2021). However, the studies mentioned above utilized similar sample sizes per treatment group and reported differences in BA excretion between dogs consuming GF, pulse-inclusive diets, and those on grain-inclusive diets: 8 dogs (Mansilla et al., 2019), and 6 dogs (Reilly et al., 2021).

In conclusion, large-breed dogs fed diets containing up to 45% whole pulses, that exceed minimum AAFCO nutrient recommendations for adult dogs did not have increased fecal BA excretion. However, dogs fed diets with 30% whole pulses and higher pea starch content had greater concentrations of propionic acid and lithocholic acid in their fecal samples compared to those fed diets with 0% whole pulses and less pea starch. Greater inclusion of pea starch may have increased the dietary amylose-to-amylopectin content, leading to microbiome alterations that occur independently of the effects of whole pulse ingredients or the total fiber composition. Further investigation of the effects of dietary amylose-to-amylopectin ratios on nutrient digestibility, gut microbiota, hind-gut fermentation, and BA metabolism in dogs is warranted. Nonetheless, dogs consuming diets with 0% or 45% inclusion of whole pulse ingredients, when macronutrients are otherwise balanced, with the same inclusion of pea starch, had similar BA excretion after 20 wk regardless of differences in total dietary fiber content. Overall, these findings support that up to 45% inclusion of whole pulses can be safely incorporated into canine diets with minimal impact on BA excretion while having potential beneficial implications to gut health, elucidated by greater propionic acid concentrations in fecal samples compared to dogs consuming a pulse-free diet.

## Supplementary Material

skaf075_suppl_Supplementary_Tables

## Abbreviations

AA: amino acid
AAFCO: Association of American Feed Control Officials
BA: bile acid
BCFA: branched-chain fatty acid
HPLC: high-performance liquid chromatography
GF: grain-free
SAA: sulfur amino acid
SCFA: short-chain fatty acid
Tau: Taurine
