# Supplementary material for: Whole pulse ingredient inclusion in macronutrient-balanced diets increased fecal concentrations of propionic acid but not total bile acids in healthy adult large-breed dogs after 20 weeks
Source: J Anim Sci. 2025 Mar 11;103:skaf075. doi: 10.1093/jas/skaf075 (PMC12019969; doi:10.1093/jas/skaf075)
Supplement: skaf075_suppl_Supplementary_Tables [file skaf075_suppl_supplementary_tables.docx]

**Supplementary Table 1: Concentration of bile acids in fecal samples from healthy adult dogs consuming one of four experimental diets with 0, 15, 30 or 45% whole pulse ingredients (Ctl, Pulse15, Pulse30, Pulse45) after 20 weeks of consumption, presented as least square means.**

| Bile Acid | Treatment | | | | | | | | |
| --- | --- | --- | --- | --- | --- | --- | --- | --- | --- |
| (µmol/g) | Ctl | SEM | Pulse15 | SEM | Pulse30 | SEM | Pulse45 | SEM | P-value |
| *n* | *7* |  | *6* |  | *7* |  | *6* |  |  |
| Primary BA^1^ |  |  |  |  |  |  |  |  |  |
| Murocholic acid | 0.0034 | 0.0012 | 0.0078 | 0.0030 | 0.0078 | 0.0028 | 0.0044 | 0.0017 | 0.2986 |
| Hyocholic acid | 0.0007^b^ | 0.0003 | 0.0041^a^ | 0.0018 | 0.0041^a^ | 0.0017 | 0.0026^ab^ | 0.0012 | 0.0187 |
| *Total primary BA* | 0.1497 | 0.0864 | 0.3736 | 0.2368 | 0.3914 | 0.2258 | 0.3132 | 0.1985 | 0.6351 |
| Secondary BA |  |  |  |  |  |  |  |  |  |
| Allocholic acid | 0.015 | 0.006 | 0.028 | 0.011 | 0.026 | 0.010 | 0.019 | 0.008 | 0.6561 |
| 7-Ketolithocholic acid | 0.187 | 0.061 | 0.492 | 0.172 | 0.339 | 0.110 | 0.209 | 0.073 | 0.1890 |
| Hyodeoxycholic acid | 0.071 | 0.020 | 0.178 | 0.054 | 0.139 | 0.039 | 0.082 | 0.025 | 0.1127 |
| Ursodeoxycholic acid | 0.020 | 0.008 | 0.059 | 0.026 | 0.040 | 0.018 | 0.018 | 0.008 | 0.2011 |
| omega-Muricholic acid | 0.0010^b^ | 0.0003 | 0.0035^a^ | 0.0011 | 0.0010^b^ | 0.0003 | 0.0020^ab^ | 0.0006 | 0.0259 |
| 3-Oxocholic acid | 0.0010 | 0.0006 | 0.0013 | 0.0009 | 0.0011 | 0.0007 | 0.0008 | 0.0006 | 0.9729 |
| Apocholic acid | 0.0192 | 0.0092 | 0.0833 | 0.0435 | 0.0781 | 0.0374 | 0.0465 | 0.0243 | 0.1602 |
| Dioxolithocholic acid | 0.0151 | 0.0044 | 0.0158 | 0.0049 | 0.0147 | 0.0043 | 0.0099 | 0.0031 | 0.6897 |
| *Total secondary BA* | 4.705 | 1.393 | 13.114 | 4.205 | 10.143 | 3.003 | 6.679 | 2.142 | 0.1209 |
| Sulfonated BA | 0.004^b^ | 0.001 | 0.014^a^ | 0.004 | 0.011^ab^ | 0.003 | 0.007^ab^ | 0.002 | 0.0418 |
| Glucuronidated BA | 0.000 | 0.000 | 0.001 | 0.000 | 0.001 | 0.000 | 0.001 | 0.000 | 0.395 |
| Glycine-conjugated BA | 0.001 | 0.000 | 0.004 | 0.001 | 0.005 | 0.002 | 0.004 | 0.001 | 0.058 |
| Taurine-conjugated BA | 0.031 | 0.015 | 0.066 | 0.034 | 0.115 | 0.054 | 0.096 | 0.049 | 0.261 |
| Total BA^*^ | 9.422 | 2.911 | 14.774 | 3.149 | 13.683 | 2.911 | 11.106 | 3.149 | 0.5898 |

n = number of observations

^a,b^Different letters within the same row indicate statistical significance (P < 0.05).

^*^Total BA represents all BA reported in this table and Table 3.

^1^BA=bile acids

**Supplementary Table 2: Proportion of bile acids from total fecal concentrations in healthy adult dogs consuming one of four experimental diets with 0, 15, 30 or 45% whole pulse ingredients (Ctl, Pulse15, Pulse30, Pulse45), after 20 weeks of consumption, presented as least square means.**

| Bile acid | Treatment | | | | | | | | |
| --- | --- | --- | --- | --- | --- | --- | --- | --- | --- |
| %, of total BA^1^ | Ctl | SEM | Pulse15 | SEM | Pulse30 | SEM | Pulse45 | SEM | P-value |
| *n* | *7* |  | *6* |  | *7* |  | *6* |  |  |
| Primary BA |  |  |  |  |  |  |  |  |  |
| Murocholic acid | 0.116 | 0.017 | 0.143 | 0.025 | 0.144 | 0.023 | 0.124 | 0.021 | 0.705 |
| Hyocholic acid | 0.014 | 0.003 | 0.029 | 0.009 | 0.038 | 0.010 | 0.031 | 0.009 | 0.081 |
| *Total primary acids* | 4.550 | 2.570 | 3.267 | 1.923 | 3.795 | 1.938 | 5.412 | 3.397 | 0.934 |
| Secondary BA |  |  |  |  |  |  |  |  |  |
| Allocholic acid | 0.307 | 0.054 | 0.224 | 0.047 | 0.231 | 0.041 | 0.244 | 0.050 | 0.633 |
| 7-Ketolithocholic acid | 2.986 | 0.700 | 3.558 | 0.916 | 2.991 | 0.604 | 2.159 | 0.594 | 0.677 |
| Hyodeoxycholic acid | 0.990 | 0.311 | 1.296 | 0.420 | 1.190 | 0.333 | 0.826 | 0.290 | 0.789 |
| Ursodeoxycholic acid | 0.382 | 0.149 | 0.412 | 0.154 | 0.325 | 0.126 | 0.233 | 0.088 | 0.724 |
| omega-Muricholic acid | 0.018 | 0.007 | 0.027 | 0.011 | 0.009 | 0.003 | 0.023 | 0.009 | 0.218 |
| 3-Oxocholic acid | 0.024 | 0.013 | 0.011 | 0.007 | 0.010 | 0.005 | 0.012 | 0.007 | 0.670 |
| Apocholic acid | 0.438 | 0.129 | 0.640 | 0.219 | 0.707 | 0.206 | 0.626 | 0.208 | 0.692 |
| Dioxolithocholic acid | 0.304^a^ | 0.037 | 0.117^b^ | 0.017 | 0.144^b^ | 0.019 | 0.129^b^ | 0.018 | 0.001 |
| *Total secondary acids* | 92.093 | 11.531 | 96.169 | 7.786 | 90.633 | 10.633 | 83.481 | 6.013 | 0.674 |
| Sulfonated BA | 0.081 | 0.017 | 0.100 | 0.024 | 0.093 | 0.019 | 0.084 | 0.020 | 0.903 |
| Glucuronidated BA | 0.009 | 0.004 | 0.004 | 0.002 | 0.010 | 0.003 | 0.007 | 0.003 | 0.582 |
| Glycine-conjugated BA | 0.027 | 0.011 | 0.023 | 0.011 | 0.048 | 0.020 | 0.045 | 0.021 | 0.604 |
| Taurine-conjugated BA | 0.625 | 0.295 | 0.354 | 0.163 | 1.044 | 0.408 | 1.946 | 1.058 | 0.383 |

n = number of observations

^a,b^Different letters within the same row indicate statistical significance (P < 0.05).

^1^BA=bile acids
